# A hybrid Spiking Neural Network–Transformer architecture for motor imagery and sleep apnea detection

**DOI:** 10.3389/fnins.2025.1716204

**Published:** 2025-12-12

**Authors:** Duc Thien Pham, Maryam Khoshkhooy Titkanlou, Roman Mouček

**Affiliations:** Department of Computer Science and Engineering, Faculty of Applied Sciences, University of West Bohemia in Pilsen, Pilsen, Czechia

**Keywords:** motor imagery, brain-computer interface, sleep apnea, EEG, ECG, spiking neural network, Transformer

## Abstract

**Introduction:**

Motor imagery (MI) classification and sleep apnea (SA) detection are two critical tasks in brain-computer interface (BCI) and biomedical signal analysis. Traditional deep learning models have shown promise in these domains, but often struggle with temporal sparsity and energy efficiency, especially in real-time or embedded applications.

**Methods:**

In this study, we propose SpiTranNet, a novel architecture that deeply integrates Spiking Neural Networks (SNNs) with Transformers through Spiking Multi-Head Attention (SMHA), where spiking neurons replace standard activation functions within the attention mechanism. This integration enables biologically plausible temporal processing and energy-efficient computations while maintaining global contextual modeling capabilities. The model is evaluated across three physiological datasets, including one electroencephalography (EEG) dataset for MI classification and two electrocardiography (ECG) datasets for SA detection.

**Results:**

Experimental results demonstrate that the hybrid SNN-Transformer model achieves competitive accuracy compared to conventional machine learning and deep learning models.

**Discussion:**

This work highlights the potential of neuromorphic-inspired architectures for robust and efficient biomedical signal processing across diverse physiological tasks.

## 1 Introduction

Sleep apnea (SA) is a common sleep disorder marked by recurrent pauses in breathing during sleep. These pauses, known as apneas, can happen repeatedly throughout the night, resulting in disrupted sleep and potentially serious health issues if not properly managed. There are two main types of SA: obstructive sleep apnea (OSA) and central sleep apnea (CSA). OSA, the more prevalent type, is usually caused by the relaxation of throat muscles, whereas CSA is due to the brain's failure to send appropriate signals to the muscles that control breathing (Benjafield et al., 2019; Kapur et al., 2017). SA is typically diagnosed by measuring the number of apnea and hypopnea events during sleep, averaged per hour to calculate indices like the Apnea-Hypopnea Index (AHI) or Respiratory Disturbance Index (RDI). These indices help determine the severity of the disorder. While direct measurements of airflow and respiratory effort, such as those obtained using an esophageal balloon, offer accuracy, they are invasive and are rarely used. Instead, polysomnography, a less intrusive and widely accepted method, is commonly preferred (American Academy of Sleep Medicine, 1999).

Electrocardiography (ECG) has gained attention as a valuable method for SA detection due to its non-invasive nature and broad accessibility. The key principle behind ECG-based SA detection is that apnea events cause alterations in heart rate variability (HRV), which can be captured using a single-lead ECG (Faust et al., 2021; Yang et al., 2022). These events disrupt airflow and blood oxygen levels, triggering compensatory cardiovascular responses. To detect SA, researchers extract informative features, such as RR intervals and statistical HRV measures from ECG signals and feed them into machine learning (ML) or deep learning (DL) models, including convolutional neural networks (CNNs). Although ECG offers a practical and affordable approach, it faces challenges like interference from coexisting cardiac conditions and the need for validation across diverse populations (Wang et al., 2019). Furthermore, balancing model accuracy with computational efficiency remains a critical hurdle, particularly for real-time or home-based healthcare applications.

Brain–computer interfaces (BCIs) are artificial systems that capture, process, and convert neural activity into control signals for external devices, facilitating direct connection between the brain and machines independent of the peripheral nervous system (Schirrmeister et al., 2017). These systems have important applications in assistive technology, neurorehabilitation, and helping people with motor disabilities regain their sensory-motor abilities. Electroencephalography (EEG) is the most widely used brain acquisition method in BCI research, as it is non-invasive, portable, offers high temporal resolution, and is affordable (Saha et al., 2021). Specific protocols and paradigms must be selected to implement an EEG-based BCI system for a particular application. The motor imagery (MI) paradigm enables users to control systems by imagining the movements of their limbs without actually executing them (Altaheri et al., 2023).

MI is a significant paradigm in BCI applications, as it enables precise intention identification when brain–computer interface (BCI) technology is used to analyze MI signals. The development of BCI devices to help people with movement disabilities (Scherer et al., 2015), assist stroke patients in their rehabilitation (Pichiorri et al., 2015), and improve motor abilities (Moran et al., 2012) depends significantly on the classification of MI signals. Deep learning networks with a neuroscience focus have recently gained popularity in brain-inspired intelligence due to their remarkable biological fidelity compared to conventional machine learning methods for MI-BCI classification.

Although significant progress has been made in automated detection of SA and MI classification, existing models often face challenges in effectively capturing both fine-grained temporal dynamics and long-range dependencies in physiological signals. In this study, we propose SpiTranNet, a Spiking-Transformer network designed for two key tasks: the automatic detection of SA using single-lead ECG signals and MI classification using multi-channel EEG data. The SpiTranNet integrates SNNs and Transformers through Spiking Multi-Head Attention (SMHA). The Transformer component provides global contextual modeling through multi-head self-attention, capturing long-range dependencies across physiological signals. The SNN component enhances this through biologically plausible spiking mechanisms within the attention layers, processing temporal dynamics and sparse, energy-efficient computations. By integrating both components, SpiTranNet aims to enhance classification performance while maintaining computational efficiency, making it a promising approach for real-time and resource-constrained biomedical applications. The main contributions of this study are as follows:

- To develop a novel hybrid neural network model named SpiTranNet for sleep apnea detection using single-lead ECG signals and motor imagery classification using multi-channel EEG data.
- To evaluate SpiTranNet's performance against existing methods and demonstrate its ability to achieve state-of-the-art accuracy across both tasks.
- To design an efficient architecture that balances model complexity and computational cost, ensuring suitability for real-time and resource-constrained healthcare applications.

The rest of the paper is organized as follows. Section 2 reviews related work. Section 3 describes the proposed model, the dataset, data preprocessing, and evaluation methodology. Section 4 presents the experimental results in both tasks, while Section 5 provides the discussion. Finally, Section 6 concludes the paper.

## 2 Related work

### 2.1 Sleep apnea

SA detection has primarily been studied using two public datasets: PhysioNet Apnea-ECG and UCDDB, with performance typically evaluated at both the per-segment and per-recording levels. Currently, models for detecting SA using single-lead ECG signals are being developed using both ML and DL approaches. Sharma et al. (2018) introduced a method utilizing a biorthogonal antisymmetric wavelet filter bank (BAWFB) combined with a support vector machine (SVM) for OSA classification. Their approach achieved average classification metrics of 90.11% accuracy, 90.87% sensitivity, 88.88% specificity, and an F-score of 0.92 on the PhysioNet Apnea-ECG dataset. Hassan and Haque (2017) applied the tunable-Q factor wavelet transform (TQWT) to extract features from single-lead ECG signals and utilized the random under-sampling boosting (RUSBoost) algorithm for SA classification. Their method achieved an accuracy of 88.88%, a sensitivity of 87.58%, and a specificity of 91.49% on the PhysioNet Apnea-ECG dataset. When evaluated on the UCDDB dataset, the performance improved, yielding 91.94% accuracy, 90.35% sensitivity, and 92.67% specificity.

Li et al. (2018) proposed a SA detection method combining a sparse autoencoder for unsupervised ECG feature learning with SVM classification, followed by a hidden Markov model (HMM) for sequence modeling. The approach achieved 85% accuracy for per-segment and 100% for per-recording detection on the PhysioNet Apnea-ECG dataset. Chen et al. (2022) proposed an end-to-end spatio-temporal model for SA detection, composed of repeated blocks combining CNN, max-pooling, and bidirectional GRU (BiGRU) layers. This architecture effectively captures both spatial morphology and temporal dynamics from ECG signals. Their CNN-BiGRU model achieved 91.22% accuracy for per-minute and 97.10% for per-recording classification on the PhysioNet Apnea-ECG dataset, as well as 91.24% accuracy on the UCDDB dataset. In other work, Tyagi and Agrawal (2023) introduced a fine-tuned enhanced Deep Belief Network (FT-EDBN) for SA classification using single-lead ECG signals. The model learns discriminative features from training data to distinguish between apnea and normal episodes. On the PhysioNet Apnea-ECG dataset, FT-EDBN achieved 89.11% accuracy for per-segment detection, with 92.28% specificity, 83.89% sensitivity, and an F1-score of 0.913. For per-recording detection, it reached 97.17% accuracy and a correlation coefficient of 0.938.

Recently, Zhao Y. et al. (2024) proposed a dual-multiscale interactive attention-based CNN (DM-IACNN) for automatic SA detection using single-lead ECG signals. The model incorporates an interactive multiscale extraction (IMSE) module to capture intra-segment features, followed by a temporal-wise attention module to enhance temporal representation. It also utilizes three adjacent ECG segments of varying lengths as multiscale inputs, fused via a scale-wise attention module to capture transition patterns across segments. Evaluated on the PhysioNet Apnea-ECG dataset, DM-IACNN achieved 91.02% accuracy for per-segment and 100% for per-recording classification. Gayen et al. (2025) proposed SmartMatch, a semi-supervised learning (SSL) framework that reduces dependence on annotated data by effectively leveraging unlabeled ECG signals. Inspired by hierarchical leader-follower dynamics, the framework combines deep metric learning with adaptive batch hard mining, pseudo-labeling, and temporal ensembling to enhance feature quality and learning stability. On the PhysioNet Apnea-ECG dataset, SmartMatch achieved per-segment results with 91.99% accuracy, 91.98% precision, 91.99% recall, and a 91.97% F1-score. Ullah et al. (2025) proposed a DL model, CSAC-Net (Convolutional Self-Attention with Adaptive Channel-Attention Network), to tackle key challenges in SA detection. The model addresses the first challenge by incorporating a convolutional self-attention module within a multi-scale projection framework, enabling the capture of long-range dependencies through diverse feature fusion. CSAC-Net was evaluated on two public datasets, PhysioNet Apnea-ECG and national sleep research resource best apnea interventions in research (NSRR-BestAIR), achieving accuracies of 93.4% and 76.1%, respectively. However, most existing approaches still rely on CNN–RNN or CNN–attention architectures that struggle to fully capture both long-range temporal dependencies and temporal dynamics in apnea-related ECG patterns. Their computational cost also limits deployment on embedded medical devices. In contrast, our SpiTranNet model offers a promising direction by deeply integrating Spiking Neural Networks and Transformers through SMHA, combining energy-efficient temporal processing with global contextual modeling to address key gaps in current SA detection research.

### 2.2 Motor imagery

The BCI Competition IV 2a dataset is one of the most popular benchmark datasets for evaluating and comparing BCI classification methods due to its well-defined multi-class MI EEG recordings. This section highlights recent studies that classified this dataset using DL techniques, highlighting the advancements in architectures and performance trends in this area. CNNs have drawn much attention due to the remarkable advances in computer hardware technology, and they have made it easier to apply deep learning to the classification of motor imaging signals (Altaheri et al., 2023). Amin et al. (2021) present an attention-guided Inception CNN and LSTM for the classification of EEG MI in rehabilitation applications. The model maintains a low computational cost while capturing temporal interdependence and multi-scale spatial features. It outperformed several modern methods (Fadel et al., 2020; Lawhern et al., 2018; Sakhavi et al., 2015) with an accuracy of 82.8% on the BCI Competition IV-2a dataset. Altaheri et al. (2022) offer ATCNet, a physics-informed attention-based Temporal Convolutional Network for EEG MI classification. The model efficiently captures spatial and temporal EEG patterns by combining CNN feature encoding, multi-head self-attention, and TCN layers. ATCNet outperformed various state-of-the-art techniques (Ingolfsson et al., 2020) with an average accuracy of 85.38% when implemented on the BCI Competition IV-2a dataset. Due to their strong feature extraction capabilities, CNN-based deep learning models have dominated EEG decoding; although they have low energy efficiency, redundant computation, and limited biological interpretability. SNNs, on the other hand, are a desirable substitute for next-generation BCIs because they process data using discrete spike events, which naturally capture temporal information in EEG while enabling energy-efficient neuromorphic deployment.

SNNs have been considered the third-generation neural network model in recent years (Maass, 1997), and they function more like biological neurons in the brain than artificial neural networks (ANNs). Its unique coding processes and rich neurodynamic features in the spatiotemporal domain have drawn considerable interest from academics. In the field of pattern recognition, SNNs are currently the subject of numerous applications, including data processing (Rasteh et al., 2022) and image recognition (Fang et al., 2021). SCNet, a spiking neural network model, was introduced by Liao et al. (2023). It combines the biological interpretability of SNNs with the feature extraction capabilities of CNNs. The model employs adaptive, learnable coding to minimize information loss, improve classification accuracy, and closely replicate neural dynamics. By using surrogate gradient learning to address SNN training issues, it achieved 88.2% accuracy on BCI IV-2a. The SNN model improved local feature extraction but has limited ability to capture global temporal dependencies between EEG channels due to their convolutional structure. Lately, Li et al. (2024) present HR-SNN, a robust SNN that uses a hybrid response spiking module to improve frequency perception and enhances spike encoding with parameter-wise gradient descent. The SNN output consumption is optimized using a diff-potential spiking decoder. HR-SNN attains an average accuracy of 77.58% on BCI Competition IV 2a, 74.95% with subject-specific transfer learning, and 67.24% on PhysioNet with global training. Despite these advancements, HR-SNN lacked attention-based procedures to capture global contextual interactions across channels and time segments and continued to use locally connected spiking modules. On the other hand, our proposed SpiTranNet integrates the attention mechanism of Transformers with the energy-efficient temporal processing of SNNs through SMHA, allowing the model to jointly learn long-range and local temporal correlations from EEG signals.

## 3 Materials and method

Conventionally, ECG and EEG signals are time series characterized by strong long-term temporal dependencies. Our proposed SpiTranNet architecture deeply integrates SNN and Transformer components through SMHA to effectively capture these dependencies while leveraging the distinct advantages of each approach. SNNs provide biologically plausible temporal processing and energy-efficient computations, while Transformers excel at learning complex, long-range temporal patterns through global contextual modeling. By integrating these complementary strengths, our model enhances classification performance and computational efficiency across diverse datasets. This hybrid framework demonstrates versatility in handling various physiological signals, showing strong potential for both SA detection from ECG and MI classification from EEG data.

### 3.1 SpiTranNet method

**Figure 1:**
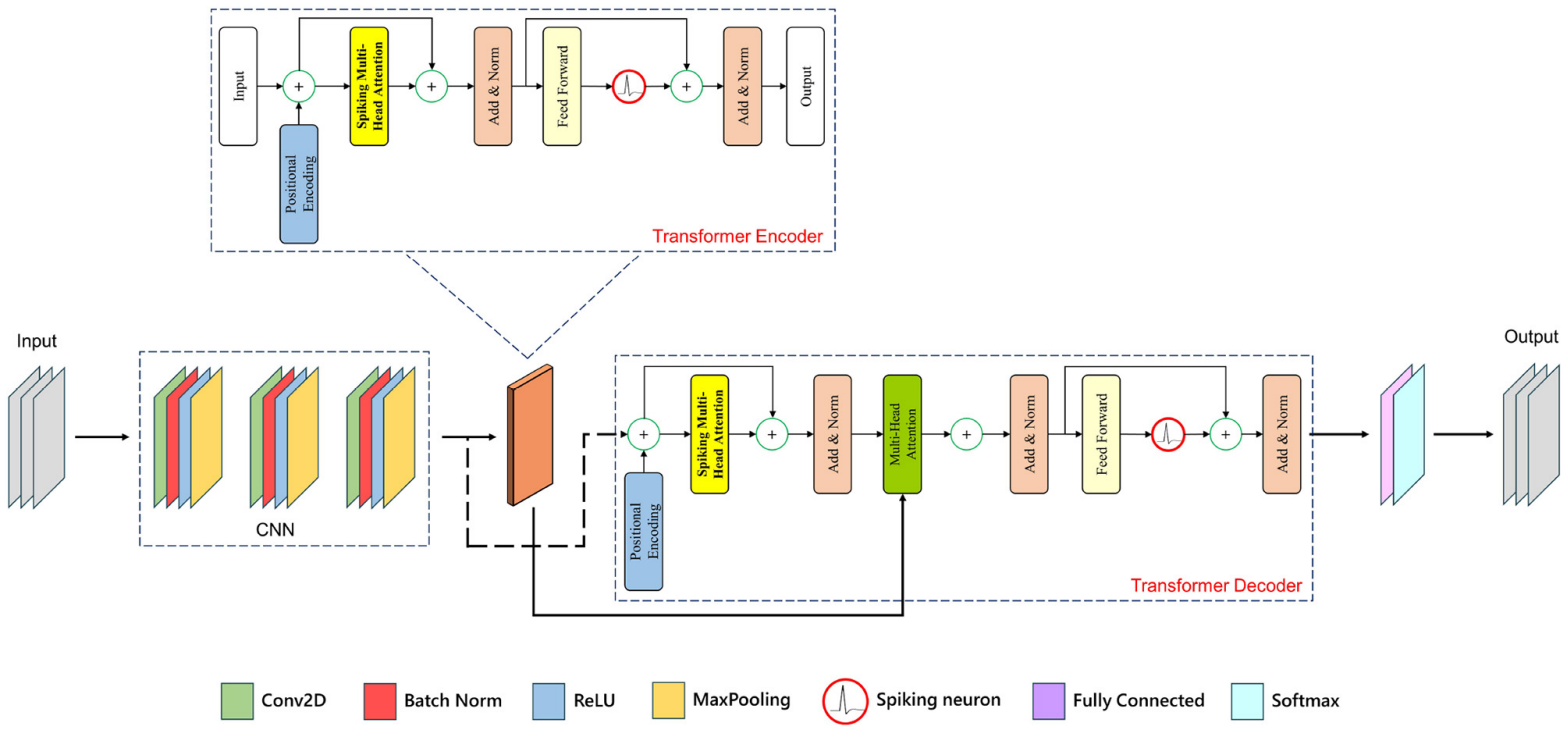
The architecture of the SpiTranNet model.

We introduce SpiTranNet, a Spiking-Transformer network for SA and MI classification. The model begins with a three-layer CNN using filter sizes of 32, 64, and 64 with a kernel size of 17, which extracts local temporal features and reduces input dimensionality through max pooling. This design captures meaningful patterns while lowering computational cost. The CNN output is then processed by a Spiking-Transformer that integrates SMHA to model temporal dependencies in EEG/ECG signals. The overall framework is illustrated in Figure 1.

The Transformer Encoder (Vaswan et al., 2017) applies layer normalization and positional encoding, then uses SMHA to capture temporal patterns with spiking dynamics. The attention output is normalized and processed through a feed-forward network that includes spiking neuron cells, enabling energy-efficient computation through sparse activations. The Transformer Decoder follows a similar structure, with SMHA and an additional attention layer to incorporate encoder outputs. Dropout is applied to prevent overfitting, creating a cohesive spiking-transformer architecture for temporal sequence processing.

#### 3.1.1 Spiking neuron

The Spiking Neuron Layer mimics the behavior of biological neurons by using threshold-based activation, generating discrete spikes when the input surpasses a certain threshold. To enable backpropagation despite the non-differentiability of spike generation, a surrogate gradient method is employed for approximating the gradient. The spiking neuron algorithm is outlined as follows (Eshraghian et al., 2023):

The output spike *S*(*t*) can be approximated as:


$$\begin{array}{l} S\left(t\right)\approx \sigma \left(V\left(t\right)\right)=\frac{1}{1+\exp\left(-\text{temp}\cdot \left(V\left(t\right)-\text{threshold}\right)\right)} \end{array}$$ (1)

where σ(*V*(*t*)) is the smoothed firing probability, temp is a temperature parameter controlling the sharpness of the sigmoid, *V*(*t*) is the membrane potential at time step *t*.

To enable smooth gradient flow during backpropagation, the gradient of the spike output with respect to the membrane potential is approximated using a sigmoid function:


$$\begin{array}{l} \frac{\partial S\left(V\left(t\right)\right)}{\partial V\left(t\right)}=\sigma \left(V\left(t\right)-\text{threshold}\right)\cdot \left(1-\sigma \left(V\left(t\right)-\text{threshold}\right)\right)\cdot \text{temp} \end{array}$$ (2)

where $\sigma \left(x\right)=\frac{1}{1+e^{-x}}$: Sigmoid function, temp: Temperature parameter controlling the steepness of the sigmoid.

#### 3.1.2 Spiking multi-head attention

In this section, we propose the SMHA mechanism, which extends traditional self-attention by integrating spiking activations. This allows attention outputs to exhibit discrete, event-driven behavior analogous to biological neurons, potentially improving energy efficiency and temporal pattern recognition.

In SMHA, the input tensors (query *Q*, key *K*, and value *V*) are linearly transformed and used to compute attention scores. The core operation involves the scaled dot-product of the queries and key, followed by a softmax function to generate attention weights. The final output is a weighted sum of *V*, emphasizing features relevant to the query:


$$\begin{array}{l} \text{Attention}\left(Q,K,V\right)=\text{softmax}\left(\frac{QK^{T}}{\sqrt{d_{k}}}\right)V \end{array}$$ (3)

where *QK*^*T*^ is the dot product between the query and key matrices, *d*_*k*_ is the dimensionality of the key vectors, softmax is applied to each row of *QK*^*T*^ to produce a set of attention scores.

The output of the SMHA mechanism is then passed through a Spiking Neuron layer.


SMHA=S(Attention(Q,K,V)) (4)

### 3.2 Dataset

In this study, we used three public datasets:

#### 3.2.1 PhysioNet Apnea-ECG dataset

The PhysioNet Apnea-ECG dataset, provided by Philipps University (Penzel et al., 2000; Goldberger et al., 2000), is a publicly available resource used to evaluate our proposed method against existing approaches. It consists of 70 single-lead ECG recordings sampled at 100 Hz, each lasting between 401 and 578 min. The dataset is split into a released set (35 recordings) for model training and parameter tuning, and a withheld set (35 recordings) for testing. According to American Academy of Sleep Medicine (AASM) standards, the withheld set includes 23 recordings from SA subjects and 12 from normal subjects.

Each one-minute segment was annotated by an expert: segments with apnea events were labeled SA, and those without as normal. The annotations do not differentiate between hypopnea and apnea and exclude CSA events. The released and withheld sets contain 17,125 (6,514 SA; 10,611 normal) and 17,303 (6,552 SA; 10,751 normal) labeled segments, respectively.

#### 3.2.2 UCD St. Vincent's University Hospital's sleep apnea database

The UCDDB dataset consists of polysomnography (PSG) recordings from 25 subjects (21 males and 4 females) (Goldberger et al., 2000). For this study, we extracted ECG signals sampled at 128 Hz, using expert-labeled annotations to classify each sleep segment as apnea or non-apnea. To mitigate class imbalance, we applied oversampling to increase the representation of apnea events in the training and validation sets. Subjects without any recorded apnea events (ucddb008, ucddb011, ucddb013, and ucddb018) were excluded from the analysis.

#### 3.2.3 BCI Competition IV 2a

**Figure 2:**
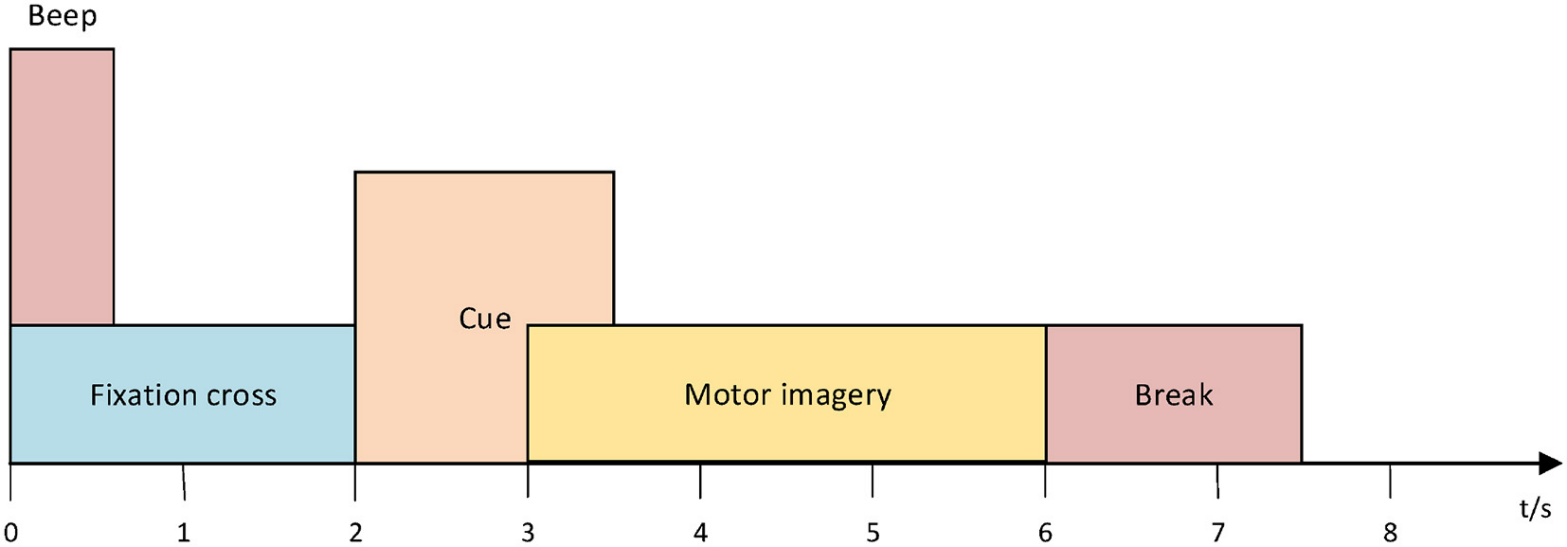
Processes within the motor imagery paradigm (Brunner et al., 2008).

The BCI Competition IV 2a (BCI-IV-2a) dataset (Brunner et al., 2008) includes recordings from nine subjects using 22 EEG channels and 3 monopolar Electrooculography (EOG) channels. This dataset is available for download at https://bbci.de/competition/iv/download/. During the experiment, subjects were instructed to imagine four types of movements: right hand, left hand, both feet simultaneously, and tongue movements. On separate dates, two recording sessions (T and E) were conducted. Each session had 288 trials, with 72 trials per class. With breaks, the average time to finish a trial was about 8 seconds, with each trial lasting roughly 6 seconds from the moment a fixed cross appeared until it vanished, as shown in Figure 2. The signals were recorded at 250 Hz and bandpass filtered between 0.5 and 100 Hz. A 50 Hz notch filter was used to reduce line noise, and the amplifier's sensitivity was adjusted to 100 μV (Tangermann et al., 2012).

### 3.3 Data preprocessing

#### 3.3.1 Sleep apnea

For the PhysioNet Apnea-ECG dataset, we used the Hamilton algorithm (Hamilton, 2002) to detect R peaks, from which R-R intervals (RRI) and R peak amplitudes were derived. ECG signals were normalized and filtered using a FIR bandpass filter. A median filter, as suggested by Chen et al. (2015), was applied to remove spikes without distorting RRI trends. Cubic interpolation was used to detect false R peaks by comparing adjacent RRIs to a robust estimate (Almutairi et al., 2021). From each 5-min segment, 900 RRI and 900 R amplitude points were extracted (Wang et al., 2019). After removing 774 faulty segments, 33,654 segments remained: 16,709 (6,473 SA, 10,236 normal) in the released set and 16,945 (6,490 SA, 10,455 normal) in the withheld set.

For the UCDDB dataset, following prior studies (John et al., 2021) that use second-by-second detection, we fixed the window size at 11 seconds, since an apnea event is defined as at least 10 seconds of breathing cessation. To effectively capture these events, overlapping 11-second windows were created with a 10-second overlap. Each window was labeled as apnea or non-apnea based on the state of the 2nd second within the window.

#### 3.3.2 Motor imagery

First, the spatial filtering is applied to the dataset. EEG recordings measure the electrical potential difference between each electrode and the reference electrode. Any noise present in the reference electrode would affect each electrode. We used Common Average Referencing (CAR) to increase the signal's signal-to-noise ratio (SNR). The new reference in CAR is the average of the electrical activity recorded across all channels. Only the exclusive activity of each EEG signal in each channel remains after the CAR filter eliminates common internal and external noise sources (Michelmann et al., 2018). The following formula can be used to determine each electrode's potential following CAR application (Yu et al., 2014):


$$\begin{array}{l} x_{i}^{\text{CAR}}\left(t\right)=x_{i}\left(t\right)-\frac{1}{C}\overset{C}{\underset{j=1}{\sum }}x_{j}\left(t\right) \end{array}$$ (5)

where *C* is the total number of electrodes, $x_{i}^{\text{CAR}}\left(t\right)$ is the spatially filtered output of electrode ith, and *x*_*j*_(*t*) is the electrical potential difference between the jth electrode and the reference.

Then we applied frequency filtering. When a movement is prepared and executed unimanually, the amplitude of the contralateral sensorimotor cortex EEG signals in the mu band (8–13 Hz) and beta band (13–30 Hz) decreases. A decrease in the amplitude of the active cortical EEG signals is referred to as event-related desynchronization (ERD). Event-related synchronization (ERS), which signifies an increase in the amplitude of the corresponding cortical signals in the resting state, occurs simultaneously with an increase in the amplitude of the ipsilateral sensorimotor cortical EEG signals in the alpha and beta frequency bands. We used a band-pass filter to extract the mu and beta bands to fully achieve ERD and ERS (Tayeb et al., 2019). In many studies, the frequency band ranges of Mu and Beta differ. For the Mu band and Beta band, respectively, [8–14 Hz] and [15–30 Hz] are typically taken into consideration (Tayeb et al., 2019).

### 3.4 Ablation study

To evaluate the contribution of each component in the proposed model, we performed ablation experiments. Ablation studies systematically analyze the impact of individual components on overall model performance under different conditions. In our experiments, we compared three models (SNN, Transformer, and SpiTranNet) by selectively removing or altering specific parts. This approach enabled us to quantify the effect of each component on performance and gain a deeper understanding of how combining spiking neurons with a Transformer architecture enhances SA and MI classification.

### 3.5 Training

The model was optimized using the Adam optimizer, employing the binary cross-entropy loss function for SA classification and the categorical cross-entropy loss function for MI classification. Based on empirical tuning, the initial learning rate was set to 0.001. We applied the ReduceLROnPlateau strategy to automatically decrease the learning rate when validation performance plateaued. To prevent overfitting, early stopping was used, terminating training if the validation loss did not improve over 30 consecutive epochs. For performance evaluation, we used five-fold cross-validation on the PhysioNet Apnea-ECG dataset. In contrast, for the UCDDB dataset, we employed a hold-out validation strategy by dividing the data into training, validation, and testing sets in an 8:1:1 ratio. For the BCI-IV-2a dataset, data were divided into training and test sets for assessment, and the evaluation session was then re-split into 80% training and 20% testing. It is worth mentioning that batch size was 16 and number of epochs were 100.

### 3.6 Performance metrics

For SA classification, we used accuracy (Acc), sensitivity (Sen), specificity (Spe), precision (Pre), F1-score, the area under the receiver operating characteristic curve (AUC), and Cohen's kappa (*k*) as the evaluation metrics for per-segment SA detection. With per-recording evaluation, the performance metrics include Acc, Sen, Spe, AUC, and the Pearson correlation coefficient (Corr). In accordance with AASM guidelines, a recording is classified as SA if the Apnea-Hypopnea Index (*AHI*) exceeds 5; otherwise, it is labeled as normal (Berry et al., 2012). The AHI for each recording is calculated from the per-segment SA detection results and is defined as follows:


$$\begin{array}{l} AHI=\frac{60}{T}\times N \end{array}$$ (6)

where *T* denotes the total number of 1-min ECG segment signals, and *N* is the number of corresponding one-minute-long SA segments.

The Pearson correlation coefficient is used to evaluate the effectiveness of the proposed method in per-recording SA detection by quantifying the correlation between the predicted and actual *AHI* values. This metric provides a reliable measure of agreement between estimated and true *AHI* values (Sharma and Sharma, 2016). It is defined as:


$$\begin{array}{l} Corr=\frac{\sum \left(X-\bar{X}\right)\left(Y-Ȳ\right)}{\sqrt{\sum {\left(X-\bar{X}\right)}^{2}\sum {\left(Y-Ȳ\right)}^{2}}} \end{array}$$ (7)

where *X* is the list of actual *AHI* values, *Y* is the list of estimated *AHI* values, $\bar{X}$ and Ȳ are mean values of *X* and *Y*, respectively.

For MI classification, the performance of our suggested model was assessed using several common metrics, which provide a thorough evaluation of the model's performance across various classification-related fields, including accuracy, precision, recall, F1-score, specificity, and *k*.

## 4 Results

### 4.1 Sleep apnea

#### 4.1.1 Results on the Physionet Apnea-ECG dataset

Table 1 summarizes the classification performance of three models for both per-segment and per-recording analysis on the Physionet Apnea-ECG dataset. Among them, SpiTranNet achieves the highest per-segment accuracy (95.0%), sensitivity (93.3%), specificity (96.0%), F1-score (0.935), AUC (0.988), and *k* (0.894). In the per-recording evaluation, SpiTranNet continues to outperform the others, attaining perfect scores in accuracy (100%), sensitivity (100%), specificity (100%), AUC (1.0), and Corr (0.999), further validating its robustness and generalization ability. The SpiTranNet model has correctly classified 23 apnea subjects and 12 normal subjects on the dataset.

**Table 1:** Per-segment and per-recording classification results on the Physionet Apnea-ECG dataset.

**Model**	**Params**	**Memory**	**Time**	**Acc**	**Sen**	**Spe**	**F1-score**	**AUC**	***k***	**Corr**
		**(%)**	**(s/epoch)**	**(%)**	**(%)**	**(%)**				
**Per-segment classification**										
SNN	231K	20	1	78.2	62.0	88.4	0.687	0.814	0.523	-
Transformer	3.8M	32	23	85.7	81.0	88.7	0.813	0.928	0.697	-
**SpiTranNet**	189K	24	5	**95.0**	**93.3**	**96.0**	**0.935**	**0.988**	**0.894**	**-**
**Per-recording classification**										
SNN	-	-	-	97.1	100	95.7	-	0.960	-	0.868
Transformer	-	-	-	94.3	91.7	95.7	-	0.992	-	0.936
**SpiTranNet**	**-**	**-**	**-**	**100**	**100**	**100**	**-**	**1**	**-**	**0.999**

The bold values indicate better results.

**Figure 3:**
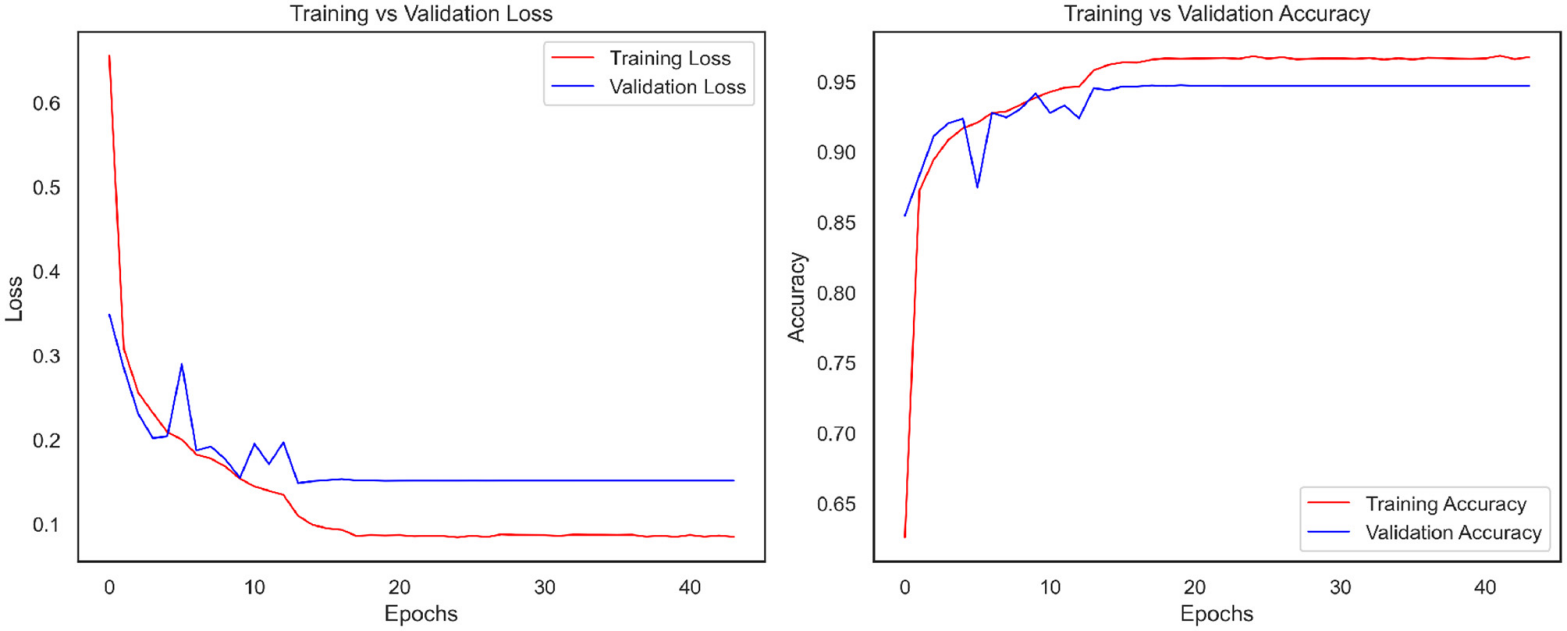
The accuracy and loss of training and validation sets on the PhysioNet Apnea-ECG dataset.

**Figure 4:**
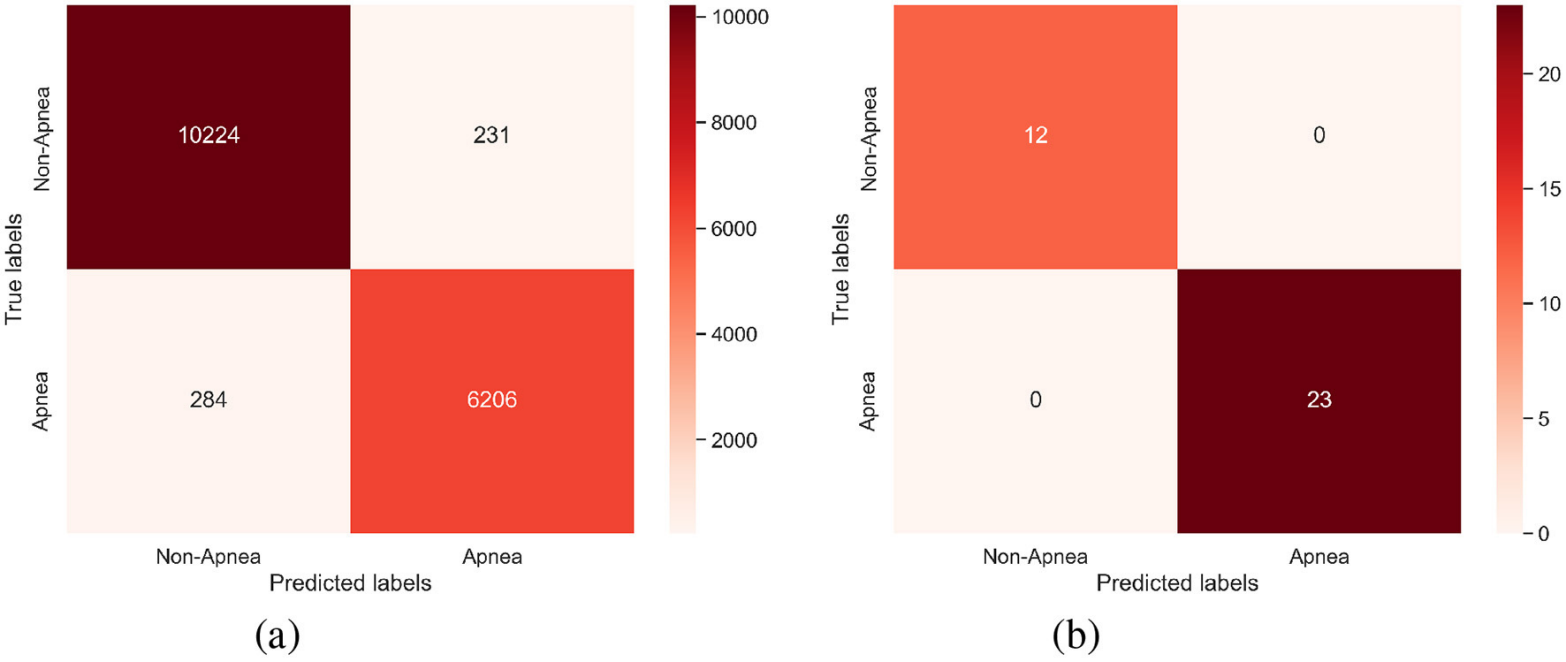
The confusion matrix of **(a)** Per-segment and **(b)** Per-recording on the PhysioNet Apnea-ECG dataset.

As shown in Tables 2, 3, the per-segment and per-recording performance comparisons on the Physionet Apnea-ECG dataset indicate that the SpiTranNet model achieves the highest accuracy among state-of-the-art methods. Figure 3 presents the training and validation accuracy and loss curves, where the model demonstrates stable performance between epochs 10 and 45, with early stopping triggered at epoch 45. The confusion matrix in Figure 4 provides the per-segment and per-recording classification outcomes.

**Table 2:** Comparison of the SpiTranNet model with existing methods for per-segment classification on the PhysioNet Apnea-ECG dataset.

**References**	**Year**	**Methods**	**Acc (%)**	**Sen (%)**	**Spe (%)**	**F1-score**	**AUC**	***k***
Sharma and Sharma (2016)	2016	LS-SVM	83.8	79.5	88.4	-	0.834	-
Li et al. (2018)	2018	DNN-HMM	84.7	88.9	82.1	0.869	0.869	-
Wang et al. (2019)	2019	LeNet-5 CNN	87.6	83.1	90.3	-	0.950	-
Almutairi et al. (2021)	2021	CNN-LSTM	90.9	91.2	90.4	0.928	-	-
Chen et al. (2022)	2022	CNN-BiGRU	91.2	86.5	94.2	0.883	-	-
Yeo et al. (2022)	2022	ResNet18	86.0	89.0	85.0	-	0.940	0.710
Chen et al. (2023)	2023	RAFNet	91.4	88.8	93.1	-	-	-
Tyagi and Agrawal (2023)	2023	FT-EDBN	89.1	83.9	92.3	0.913	0.960	-
Srivastava et al. (2023)	2023	ALexNet + LSTM	90.9	95.5	83.4	-	-	-
Abasi et al. (2023)	2023	MHBA+LeNet-5	91.3	90.1	93.6	-	0.975	-
Zhao Y. et al. (2024)	2024	DM-IACNN	91.0	84.3	95.1	0.876	-	-
Nguyen et al. (2024)	2024	MPCNN	92.1	87.7	94.8	-	-	-
Pham and Moucek (2025)	2025	CNN-Transformer	91.4	90.6	91.9	0.890	0.969	-
Ullah et al. (2025)	2025	CSAC-Net	93.4	91.4	94.7	0.914	0.980	-
Gayen et al. (2025)	2025	SmartMatch	92.0	92.0	92.0	0.920	-	-
Zhao Y. et al. (2025)	2025	SE-MSResNet	91.7	89.4	93.2	0.892	0.972	-
Cai et al. (2025)	2025	TP-CL	91.4	90.2	92.1	-	0.963	-
		**SpiTranNet**	**95.0**	**93.3**	**96.0**	**0.935**	**0.988**	**0.894**

The bold values indicate better results.

**Table 3:** Comparison of the SpiTranNet model with existing methods for per-recording classification on the PhysioNet Apnea-ECG dataset.

**References**	**Year**	**Methods**	**Acc (%)**	**Sen (%)**	**Spe (%)**	**AUC**	**Corr**
Sharma and Sharma (2016)	2016	LS-SVM	97.1	95.8	100	0.978	0.841
Li et al. (2018)	2018	DNN-HMM	100	100	100	-	-
Wang et al. (2019)	2019	LeNet-5 CNN	97.1	100	91.7	-	0.943
Chen et al. (2022)	2022	CNN-BiGRU	97.1	95.7	100	-	0.984
Yeo et al. (2022)	2022	ResNet18	97.0	98.0	96.0	0.99	-
Chen et al. (2023)	2023	RAFNet	100	100	100	-	0.985
Tyagi and Agrawal (2023)	2023	FT-EDBN	97.1	100	91.7	-	0.938
Srivastava et al. (2023)	2023	ALexNet + LSTM	97.1	-	-	-	-
Zhao Y. et al. (2024)	2024	DM-IACNN	100	100	100	-	-
Nguyen et al. (2024)	2024	MPCNN	100	100	100	-	0.989
Pham and Moucek (2025)	2025	CNN-Transformer	100	100	100	1	0.984
Zhao Y. et al. (2025)	2025	SE-MSResNet	100	100	100	-	-
Cai et al. (2025)	2025	TP-CL	100	100	100	-	-
		**SpiTranNet**	**100**	**100**	**100**	**1**	**0.999**

The bold values indicate better results.

#### 4.1.2 Results on the UCDDB dataset

**Figure 5:**
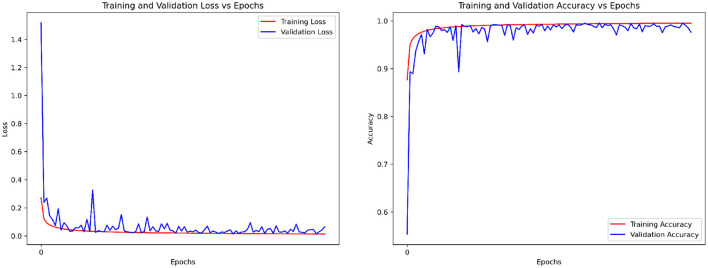
The accuracy and loss of training and validation sets on the UCDDB dataset.

Based on the results in Table 1, the SpiTranNet model was selected for evaluation on the UCDDB dataset, primarily due to its superior performance compared to other models. As shown in Table 4, SpiTranNet demonstrates better performance than existing approaches, achieving an accuracy, sensitivity, specificity, F1-score, AUC, and *k* of 99.4%, 97.6%, 99.5%, 0.899, 0.999, and 0.896, respectively. Figure 5 shows the training and validation accuracy and loss curves on the UCDDB dataset.

**Table 4:** Comparison of the SpiTranNet model with existing methods on the UCDDB dataset.

**References**	**Year**	**Methods**	**Acc (%)**	**Sen (%)**	**Spe (%)**	**F1-score**	**AUC**	***k***
Wang et al. (2019)	2019	LeNet-5 CNN	71.8	26.6	86.9	-	-	-
Mashrur et al. (2021)	2021	SCNN	81.9	71.6	86.1	0.696	-	-
Zarei et al. (2022)	2022	CNN-LSTM	93.7	90.7	95.8	-	-	-
Chen et al. (2022)	2022	CNN-BiGRU	92.3	70.5	93.9	0.760	-	-
Yeo et al. (2022)	2022	ResNet18	78.0	60.0	84.0	-	0.820	0.440
Nguyen et al. (2024)	2024	MPCNN	81.3	40.3	87.7	-	-	-
Zhao Y. et al. (2025)	2025	SE-MSResNet	84.2	88.3	81.1	-	-	-
Cai et al. (2025)	2025	TP-CL	82.7	77.9	84.9	-	0.873	-
		**SpiTranNet**	**99.4**	**97.6**	**99.5**	**0.899**	**0.999**	**0.896**

The bold values indicate better results.

### 4.2 Motor imagery

Table 5 summarizes binary MI classification (right-hand/left-hand) performance metrics of our proposed method on the BCI-IV-2a dataset, where the model achieved an accuracy of 88.4%, precision of 88.5%, recall of 89.5%, F1-score of 0.865, specificity of 83.0%, kappa of 0.750, and an AUC of 0.948. These results indicate consistent effectiveness across all evaluation measures, including accuracy, precision, recall, F1-score, and specificity, with accuracy exceeding 90% for most subjects. This demonstrates the model's robustness and potential applicability to the classification task, as well as its potential use in real-life scenarios.

**Table 5:** All subjects' classification results on the BCI-IV-2a dataset.

**Subject**	**Accuracy**	**Precision**	**Recall**	**F1-score**	**Specificity**	**Kappa**	**AUC**
1	0.965	0.966	0.966	0.966	0.964	0.930	0.982
2	0.860	0.889	0.828	0.857	0.893	0.720	0.901
3	0.982	1.000	0.966	0.982	1.000	0.965	0.996
4	0.912	0.929	0.897	0.912	0.929	0.825	0.946
5	0.772	0.727	0.857	0.787	0.690	0.545	0.764
6	0.807	0.821	0.793	0.807	0.821	0.614	0.810
7	0.965	0.964	0.964	0.964	0.966	0.930	0.984
8	0.912	0.897	0.929	0.912	0.897	0.825	0.945
9	1.000	1.000	1.000	1.000	1.000	1.000	1.000
**Average**	**0.884**	**0.885**	**0.895**	**0.865**	**0.830**	**0.750**	**0.948**

The bold values indicate better results.

**Figure 6:**
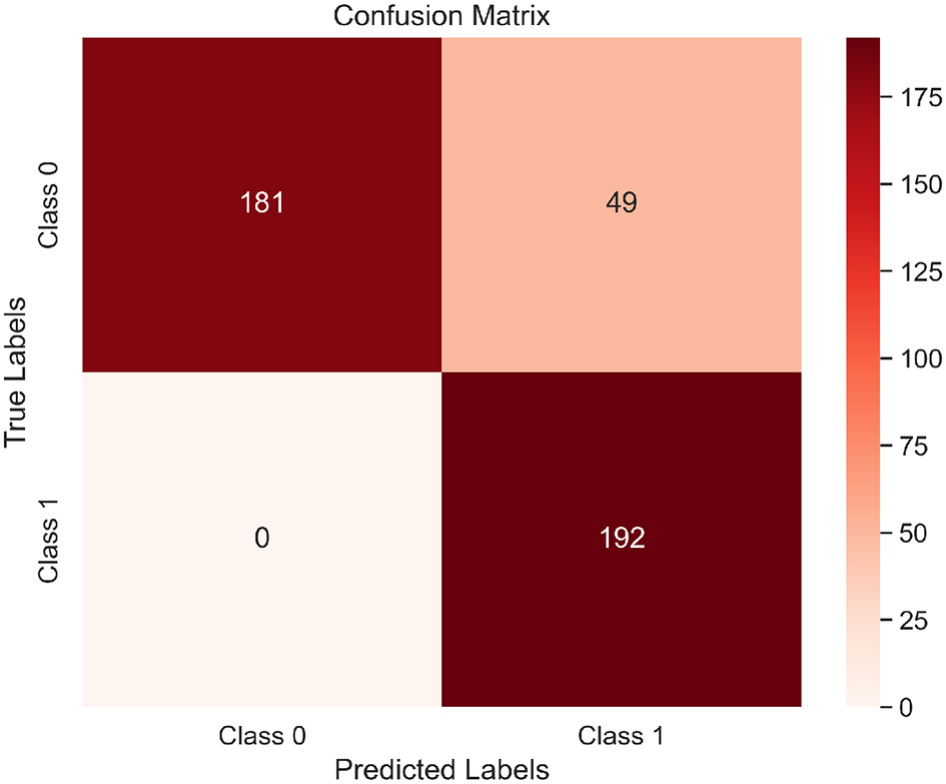
The confusion matrix plot for all subjects on the BCI-IV-2a dataset.

Table 6 compares our approach with representative state-of-the-art methods, demonstrating better accuracy and robustness compared to most existing state-of-the-art models. These results contribute to the creation of reliable, understandable, and implementable BCI systems. Additionally, the confusion matrix for all subjects is provided in Figure 6, illustrating the total counts of true positives, true negatives, false positives, and false negatives.

**Table 6:** Performance comparison with state-of-the-art methods on the BCI-IV-2a dataset.

**References**	**Year**	**Methods**	**Acc (%)**
Wei and Wei (2016)	2016	BSA-FT CSP	76.0
Amin et al. (2021)	2021	Attention-based Inception + BiLSTM	82.8
Altaheri et al. (2022)	2022	ATCNet	85.4
Liao et al. (2023)	2023	SCNet	88.2
Chowdhury et al. (2023)	2023	EEGNet fusion	74.3
Zhao W. et al. (2024)	2024	CTNet	82.5
Gu et al. (2025)	2025	CLTNet	83.0
Zhao W. et al. (2025)	2025	MSCFormer	83.0
Otarbay and Kyzyrkanov (2025)	2025	SVM-self attention	83.3
Liao et al. (2025)	2025	CIACNet	85.2
		**SpiTranNet**	**88.4**

The bold values indicate better results.

## 5 Discussion

In this study, we introduced the SpiTranNet model for classifying SA using single-lead ECG signals from the PhysioNet Apnea-ECG and UCDDB datasets, as well as for MI classification using multi-channel EEG signals from the BCI Competition IV 2a dataset. The model leverages the complementary strengths of its components: SNNs enable biologically plausible temporal processing and energy-efficient computations, whereas Transformers capture complex, long-range temporal patterns through global contextual modeling. By combining these capabilities, SpiTranNet enhances workflow efficiency and improves generalizability across different datasets. This hybrid approach demonstrates flexibility in handling a variety of physiological signals and classification tasks, making it a strong model for both SA and MI detection. We also compared its performance against existing state-of-the-art methods.

### 5.1 Sleep apnea

Table 1 compares per-segment and per-recording classification results on the PhysioNet Apnea-ECG dataset. The proposed SpiTranNet consistently outperforms both SNN and Transformer models, achieving 95.0% per-segment accuracy and perfect per-recording performance (100% accuracy, sensitivity, and specificity). While the SNN only model demonstrates the fastest training time (1 s/epoch) and lowest GPU memory usage (20%), its performance is limited (78.2% accuracy), highlighting the challenge of capturing complex temporal patterns with spiking mechanisms alone. However, when integrated into SpiTranNet through SMHA, the SNN component provides crucial benefits: it enables biologically plausible temporal processing while maintaining computational efficiency. SpiTranNet demonstrates remarkable efficiency, requiring only 189K parameters (18% fewer than SNN only and 20 × fewer than Transformer) while achieving training times of 5 seconds per epoch, substantially faster than Transformer and using only 24% GPU memory.

Tables 2, 3 further demonstrate that SpiTranNet achieves the high metrics across nearly all evaluation measures, including accuracy (95.0%), sensitivity (93.3%), specificity (96.0%), F1-score (0.935), AUC (0.988), and *k* (0.894), outperforming traditional methods such as LS-SVM Sharma and Sharma, (2016), DNN-HMM Li et al., (2018), and LeNet-5 CNN Wang et al., (2019), as well as recent deep learning approaches including DM-IACNN (Zhao Y. et al., (2024), MPCNN Nguyen et al., (2024), TP-CL Cai et al., (2025), and CSAC-Net Ullah et al., (2025). For per-recording classification, SpiTranNet achieves 100% accuracy, sensitivity, specificity, and an AUC of 1.0, with the highest correlation (0.999) between predicted and true apnea indices, demonstrating robust performance at the patient level where consistent detection across overnight recordings is crucial.

Table 4 highlights SpiTranNet's better performance on the UCDDB dataset, achieving 99.4% accuracy, 97.6% sensitivity, 99.5% specificity, F1-score of 0.899, AUC of 0.999, and kappa of 0.896. Compared to earlier models, including LeNet-5 CNN (Wang et al., 2019), SCNN (Mashrur et al., 2021), ResNet18 (Yeo et al., 2022), SE-MSResNet (Zhao Y. et al., 2025), TP-CL (Cai et al., 2025), CNN-LSTM (Zarei et al., 2022), and CNN-BiGRU (Chen et al., 2022), SpiTranNet demonstrates superior sensitivity and overall balanced performance, which is essential for minimizing missed apnea events in clinical diagnostics. These results emphasize the effectiveness of integrating SNNs and Transformers, providing a model that is both accurate and computationally efficient, with robust generalization across multiple datasets.

Overall, SpiTranNet's excellent balance between sensitivity and specificity, along with its high AUC and kappa scores, indicates both precise and reliable SA detection. Its generalization across two SA datasets (PhysioNet and UCDDB) highlights its robustness and suitability for practical, real-world applications in SA screening. Despite these encouraging results, the current validation is limited to publicly available datasets and offline evaluation, and occasional per-segment misclassifications remain, which may affect diagnostic reliability.

### 5.2 Motor imagery

Through the exploration and optimization of deep learning models across the benchmark dataset, specifically the BCI competition IV-2a, this study aimed to improve the classification of MI EEG signals. Table 5 summarizes the classification performance metrics of our proposed method on the BCI-IV-2a dataset. The best accuracy is achieved by subject 9 (100%); however, the average accuracy and precision for all subjects are 88.4% and 88.5%, respectively.

Table 6 compares the accuracy of our proposed method with that of state-of-the-art methods. Our method achieves the best classification accuracy (88.4%).

The development of MI-BCI systems can utilize the model suggested in this study. MI-BCI systems can perform very accurately if they are properly connected or combined with external devices (like sensors, feedback systems, or assistive technologies). In other words, by integrating with the right hardware or tools, these systems can more effectively interpret brain signals and enhance their performance. Although our proposed model has demonstrated superior performance in MI-EEG decoding, it still faces certain limitations, such as inter-subject variability and computational efficiency (the number of parameters was around 2M). Enhancing model generalization, extending to multi-class environments, and optimizing for real-time usage in resource-constrained scenarios should be the main goals of future work.

## 6 Conclusion

In this study, we introduced SpiTranNet, a hybrid Spiking Neural Network–Transformer architecture designed for physiological signal classification tasks, with a focus on SA detection using single-lead ECG and MI classification using multi-channel EEG. By integrating the biologically plausible temporal processing and energy-efficient computations of SNNs with the powerful long-range dependency modeling of Transformers through SMHA, SpiTranNet outperformed both standalone SNN and Transformer models across multiple benchmarks.

For SA detection, SpiTranNet achieved 95.0% per-segment accuracy and 100% per-recording accuracy on the PhysioNet Apnea-ECG dataset, along with perfect sensitivity, specificity, AUC, and *k*. On the UCDDB dataset, it attained 99.4% accuracy, with an AUC of 0.999. These results represent significant improvements over state-of-the-art models, highlighting SpiTranNet's robustness and indicating a promising direction for clinical applications. For MI classification, SpiTranNet achieved an average accuracy of 88.4% on the BCI Competition IV 2a dataset, with subject-level performance ranging up to 100% accuracy. This competitive performance further demonstrates its ability to generalize across different physiological modalities.

Future work will focus on extending SpiTranNet for real-time and embedded deployment, improving subject-independent generalization across larger and more diverse datasets, and incorporating adaptive learning strategies such as transfer learning to minimize retraining efforts. Further exploration of multimodal integration (e.g., ECG, EEG, EMG, SpO_2_, respiration) and hardware-efficient implementations on neuromorphic or low-power devices will also be critical steps toward translating SpiTranNet into practical, real-world medical and BCI applications.

Taken together, these findings demonstrate that SpiTranNet is a flexible, efficient, and highly accurate model that can generalize across multiple biomedical domains. Its ability to process both cardiac rhythms (ECG) and complex brain activity patterns (EEG) suggests its potential as a unified framework for diverse healthcare applications, ranging from sleep disorder screening to BCI systems.

## Data Availability

The original contributions presented in the study are included in the article/supplementary material, further inquiries can be directed to the corresponding author.
